# Profile of Congenital Hypothyroidism in DR. Cipto Mangunkusumo Hospital

**DOI:** 10.1186/1687-9856-2013-S1-P146

**Published:** 2013-10-03

**Authors:** Dana Nur Prihadi, Frida Soesanti, Aman B Pulungan, Bambang Tridjaja, Jose RL Batubara2

**Affiliations:** 1 Department of Paediatrics, Gatot Soebroto Central Army Hospital, Jakarta, Indonesia; 2 Department of Paediatrics, School of Medicine, University of Indonesia, dr. Cipto Mangun Kusumo Hospital, Jakarta, Indonesia

## Background

Congenital Hypothyroidism (CH) affects children from infant and results from a partial or complete loss of thyroid function. Unrecognized CH can make some problems in health of children.

## Method

a retrospective study of congenital hypothyroidism in dr. Cipto Mangunkusumo Hospital, during January 2010-December 2011.

## Results

there were 40 children with CH included in the study. Boys 12(30 %) and girls 28(70%). Age when diagnosed 0-3 months 4(10%), 3-6 months 2(5%), 6-12 months 9(22,5%), 1-6 years 20(50%), 6-14 years 5(12,5%). Clinical manifestation: delayed development 27,5%, underweight 17,5%, acute respiratory infection 15%, constipation 12,5%, hypotonia 7,5%, speech delay 5%, short stature 5%, enlarged tongue 2,5%, vomite 2,5%, icteric 2,5%, enlarged lymph node of colli 2,5%. We found suspect down syndrome 4(10%), severe malnutrition 4(10%), short stature 3(7,5%), mental retardation 2(5%).

## Conclusion

in our study, most common age of CH patients was 1-6 years, most sex was girls, which often founded were delayed development.

